# Supplementary material for: Chronological adhesive cardiac patch for synchronous mechanophysiological monitoring and electrocoupling therapy
Source: Nat Commun. 2023 Oct 6;14:6226. doi: 10.1038/s41467-023-42008-9 (PMC10558550; doi:10.1038/s41467-023-42008-9)
Supplement: Supplementary file 1 — Supplementary Information [file 41467_2023_42008_MOESM1_ESM.pdf]

Supplementary Information

Chronological adhesive cardiac patch for synchronous mechanophysiological monitoring and electrocoupling therapy

Chaojie Yu[1,2], Mingyue Shi[1,3], Shaoshuai He[1,4], Mengmeng Yao[1], Hong Sun[5]*, Zhiwei Yue[5], Yuwei Qiu[1], Baijun Liu[1], Lei Liang[1], Zhongming Zhao[1], Fanglian Yao[1,2]*, Hong Zhang[1,2]*, Junjie Li[1,2]*

*Corresponding author. Email: sunhong@ncst.edu.cn; yaofanglian@tju.edu.cn; zhanghong@tju.edu.cn; li41308@tju.edu.cn

Contents

Supplementary Methods

Supplementary Fig. 1 | FT-IR spectra of PANi, b-PANi and f-PANi.

Supplementary Fig. 2 | Doping structure of conducting polymers.

Supplementary Fig. 3 | The gelation time of the CAHP.

Supplementary Fig. 4 | The absorbance of f-PANi infiltrated the gelatin substrate.

Supplementary Fig. 5 | Photographs of CAHP adhesion to various tissues.

Supplementary Fig. 6 | The fracture energy of the CAHP.

Supplementary Fig. 7 | Design diagram of a custom applicator.

Supplementary Fig. 8 | The shear thinning and self-healing properties of the CAHP.

Supplementary Fig. 9 | The swelling performance of the CAHP.

Supplementary Fig. 10 | Rheological properties of the CAHP.

Supplementary Fig. 11 | Electrical self-healing process of the CAHP.

Supplementary Fig. 12 | Electrochemical impedance of the CAHP.

Supplementary Fig. 13 | The charge injection capacity of the PVA hydrogel and CAHP.

Supplementary Fig. 14 | The electrochemical stability of the CAHP.

Supplementary Fig. 15 | Stress−strain curves and deformation-induced current change curves of CAHP-12% in uniaxial tension.

Supplementary Fig. 16 | Stress−strain curves and deformation-induced current change curves of CAHP-12% under uniaxial compression.

Supplementary Fig. 17 | The cytocompatibility of the CAHP.

Supplementary Fig. 18 | The histocompatibility of the CAHP.

Supplementary Fig. 19 | The implantation process of the CAHP.

Supplementary Fig. 20 | Calcium transient testing of rat hearts.

Supplementary Fig. 21 | End-diastolic diameters and volumes of rat hearts.

Supplementary Fig. 22 | End-systolic diameters and volumes of rat hearts.

Supplementary Fig. 23 | Fractional shortening of rat hearts.

Supplementary Fig. 24 | Statistical analysis of myocardial retention and integrated optical density of Cx43.

Supplementary Table 1 | Semiquantitative elemental percent in f-PANi.

Supplementary Table 2 | Component content of the CAHPs.

Supplementary Movie 1 | Conducting polymer dispersion permeation through the filter membrane.

Supplementary Movie 2 | Rapid self-healing of the CAHP.

Supplementary Methods

Materials. Hydrochloric acid solution (HCl, 37%), sodium hydroxide (NaOH, 99.99%), potassium bromide (KBr, 99.99%), *N*,*N*-dimethylformamide (DMF, 99.5%), and gelatin (240 g bloom) were purchased from Aladdin. Ethylene diamine tetraacetic acid (EDTA) solution (50X, C1034), phosphate buffer solution (PBS, 10 mM, P1010), donkey serum (SL050), and RPMI 1640 medium were purchased from Solarbio.

Synthesis of PANi and b-PANi. To synthesize polyaniline (PANi), 12 mmol aniline (AN) and 12 mmol ammonium persulphate (APS) were dissolved in 100 mL of HCl solution (0.2 M) and reacted for 24 h at room temperature. Similarly, to prepare borated polyaniline (b-PANi), 4 mmol AN, 8 mmol 3-aminobenzeneboronic acid monohydrate (BAN), and 12 mmol APS were dissolved in 100 mL of HCl solution (0.2 M) and reacted for 24 h at room temperature. PANi and b-PANi were purified by several aqueous washing and centrifugation (5000 rpm, 10 min) operations to remove impurities. The PANi and b-PANi solutions were adjusted to neutral by adding an appropriate amount of NaOH solution (3 M), dialyzed for three days, and freeze-dried.

Fourier Transform Infrared spectroscopy. The chemical structures of PANi, b-PANi, and functionalized polyaniline (f-PANi) were studied by Fourier transform infrared spectroscopy (FT-IR, Nicolet 6700, Thermo Fisher, USA). The powder samples were ground and pressed into a transparent sheet with KBr. The FT-IR was tested in a range of 700−1900 cm^{-1}, with 32 scans and a resolution of 4 cm^{-1}.

X-ray photoelectron spectroscopy. Dried PANi, b-PANi, and f-PANi powders were pressed into slices and analyzed by X-ray photoelectron spectroscopy (XPS, K-Alpha+,

Thermo Fisher, USA). The peaks of C1*s* and N1*s* were separated, and elements C, N, B, and O were analyzed semiquantitatively by Avantage software.

UV−vis spectroscopy. Aqueous dispersions of PANi, b-PANi, and f-PANi of 0.5 $mg \cdot mL^{-1}$ were prepared by sonication. UV−vis spectra (Evolution 201, Thermo Fisher, USA) were detected at wavelengths of 200−900 nm.

Potential and particle size analysis. Aqueous dispersions of 0.5 $mg \cdot mL^{-1}$ PANi, b-PANi, and f-PANi were loaded into Malvern potential cells. The zeta potential and hydration particle size of the conductive polymers were detected by a Zetasizer (Nano ZS, Malvern, England).

Contact angle test. PANi, b-PANi, and f-PANi were dissolved in DMF to obtain 0.5 $g \cdot mL^{-1}$ conducting polymer solutions, which formed films after solvent evaporation. Photos of oil and water droplets were captured after 5 s of spreading on the film. The oil/water contact angles of the conducting polymers were measured using the goniometric method (JC2000FM, Zhongchen Digital Technology Apparatus, China).

Filtration ratio. The 1 wt% PANi, 1 wt% b-PANi and 12 wt% f-PANi aqueous dispersions were injected through a 0.45 μm filter membrane. The absorbance of the pre-filtered and post-filtered dispersions was measured in the wavelength range of 350 to 550 nm. The ratio of the absorbance of the filtrate to the absorbance of the original solution at 400 nm was defined as the filtration ratio.

Fabrication of polyvinyl alcohol hydrogel. Polyvinyl alcohol (PVA) powder (1 g) was dissolved in 9 mL of PBS (10 mM) at 90 °C to obtain a PVA solution with a mass fraction of 10 wt%. The PVA solution was frozen at −20 °C for 8 h and then thawed

gradually at 37 °C. Crystallization between the PVA polymer chain segments induced the formation of PVA hydrogels.

Morphology characterization. To obtain the network structure of the chronological adhesive hydrogel patch (CAHP) in the initial gel state, PVA solution was mixed with f-PANi solution and injected into liquid nitrogen within 10 seconds. The rapidly growing ice crystals quickly immobilized the initially formed network structure. In addition, the CAHP in the complete gel state was frozen after gelation was carried out for 10 min. The samples were freeze-dried and sprayed with gold. The pore structures of CAHP in the initial and complete gelation and the interfacial morphology between CAHP and myocardium were characterized by scanning electronic microscopy (SEM, S-4800, Hitachi, Japan).

Permeability. The CAHP was contacted with transparent gelatin hydrogel in the initial gel state and complete gel state for 10 min. After removal of CAHP, the gelatin hydrogels were assayed for absorbance at 400 nm, and untreated gelatin was used as a control.

Swelling tests. The swelling behaviors of the CAHP were investigated by weight analysis. The prepared CAHPs were soaked in PBS solution for 96 h and weighed at the set time. The swelling rate was calculated using the following supplementary equation (1):

$$\text{Swelling rate} = \frac{W_t - W_0}{W_0} \times 100 \quad (1)$$

where W_t and W_0 are defined as the swollen weight and original weight of the hydrogel samples, respectively.

Fracture tests. Fracture tests were performed on a tensile tester to determine the fracture energy of the CAHPs. The CAHPs were cut into a rectangular shape (40 mm × 10 mm × 2 mm) with a 5 mm initial notch at the midpoint of the long edge. The notched and un-notched specimens of the CAHPs were stretched at a rate of 20 mm·min^{-1} to record the stress (σ) and extension ratio (λ, the ratio of the stretched length to the original length). Fracture work (W) was calculated from the integral area of the $\sigma-\lambda$ curve of the un-notched samples. The fracture toughness (Γ) was calculated using the following supplementary equation (2):

$$\Gamma = 2 \times \frac{3}{\sqrt{\lambda_c}} \times W(\lambda_c) \times c \quad (2)$$

where λ_c is the extension ratio of the notched sample at the crack fracture. $W(\lambda_c)$ is the integral area of the $\sigma-\lambda$ curve for the un-notched samples in the λ_c range. c is the initial length of the crack.

Injection force measurements. The injection force of the CAHP was analyzed using a universal machine. Briefly, the CAHPs were loaded into a 1 mL plastic syringe and injected through customized brush heads. The syringe plunger was depressed using an upper compressive platen, and the syringe housing was fitted into a lower tensile grip. The injection rate was controlled by setting the speed of the compression platen. The CAHPs were squeezed out at a flow rate of 5 mL·min^{-1}. The injection force was the average extrusion force on the force–displacement curve.

Electrical self-healing test. The CAHP was connected to a circuit with a 3 V potential, and the CAHP was disconnected by the blade and reconnected. Current changes were monitored to further demonstrate the self-recovery of electrical connections within the

hydrogel networks. Electrical healing efficiency calculated by the ratio of the current value of the reconnection to the current value before disconnection.

Cytocompatibility. The cytocompatibility of CAHP was detected by acridine orange staining (CA1143, Solarbio) and the MTT assay kit (M1020, Solarbio) using L929 mouse fibroblasts (iCell-m026, iCell Bioscience). The f-PANi and PVA solutions were passed through a 0.45 μm filter for sterilization. The CAHPs (1 g) were macerated in complete medium (10 mL) at 37 °C for 48 h to obtain the hydrogel extract. Cells were seeded in a 96-well tissue culture plate (Jet Biofil) at a density of 1×10^4/well and incubated at 37 °C, 5% CO_2, and 100% humidity for 12 h. The medium was replaced with 200 μL hydrogel extract for 24 h and 48 h at 37 °C. Cells cultured in fresh medium were used as a control. Acridine orange staining and MTT detection were performed according to a standard protocol. A fluorescence microscope was used to observe cell morphology (ECLIPSE Ts2, Nikon, Japan). The absorbance at 490 nm was determined by an enzyme immunoassay (Infinite M Nano+, Tecan, Austria) in the MTT assay. Cell viability was calculated by the following supplementary equation (3):

$$\text{Cell viability} = \frac{A_{\text{sample}}-A_{\text{blank}}}{A_{\text{control}}-A_{\text{blank}}} \times 100\ \% \tag{3}$$

where A_{sample} and A_{control} represent the MTT solution absorbance after cell culture in hydrogel extract and fresh medium, respectively. A_{blank} represents the MTT solution absorbance without cell culture. Samples with relative cell viability less than 70% were considered to be cytotoxic.

Histocompatibility. Kunming mice (male, 8 weeks old, n = 3) were anesthetized with isoflurane, and 100 μL of CAHP was injected into their subcutaneous tissue. The

hydrogel−tissue was removed after 7 days of implantation, fixed with paraformaldehyde at room temperature for 24 h, and embedded in paraffin after gradient dehydration. The specimens were cut into tissue sections of 4 μm thickness and placed on slides. Tissue sections were deparaffinized and rehydrated. Subsequently, hematoxylin stained the nuclei blue, and eosin stained the cytoplasm red.

Immunofluorescence staining. The tissue slices were deparaffinized and rehydrated. Tissue sections were immersed in EDTA antigen repair buffer for 3 min under high-pressure steam to repair the antigen. After natural cooling, the sections were placed in PBS (pH 7.4) and shaken on a decolorization shaker. Then, the sections were incubated with 10% donkey serum solution at 37 °C for 2 h. A proportion of primary antibody was added to the sections and incubated overnight at 4 °C in a wet box. After several washes in PBS, the secondary antibody covered the tissue and was incubated for 2 h at 37 °C. A DAPI-containing blocker was added dropwise and incubated at room temperature for 10 min.

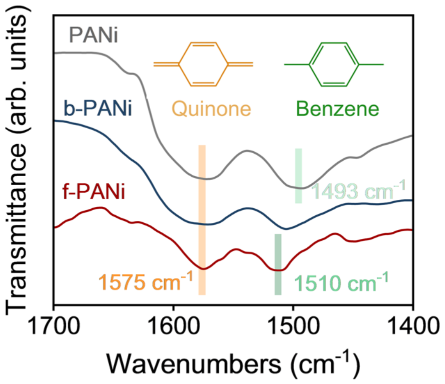

Supplementary Fig. 1 | FT-IR spectra of PANi, b-PANi and f-PANi.

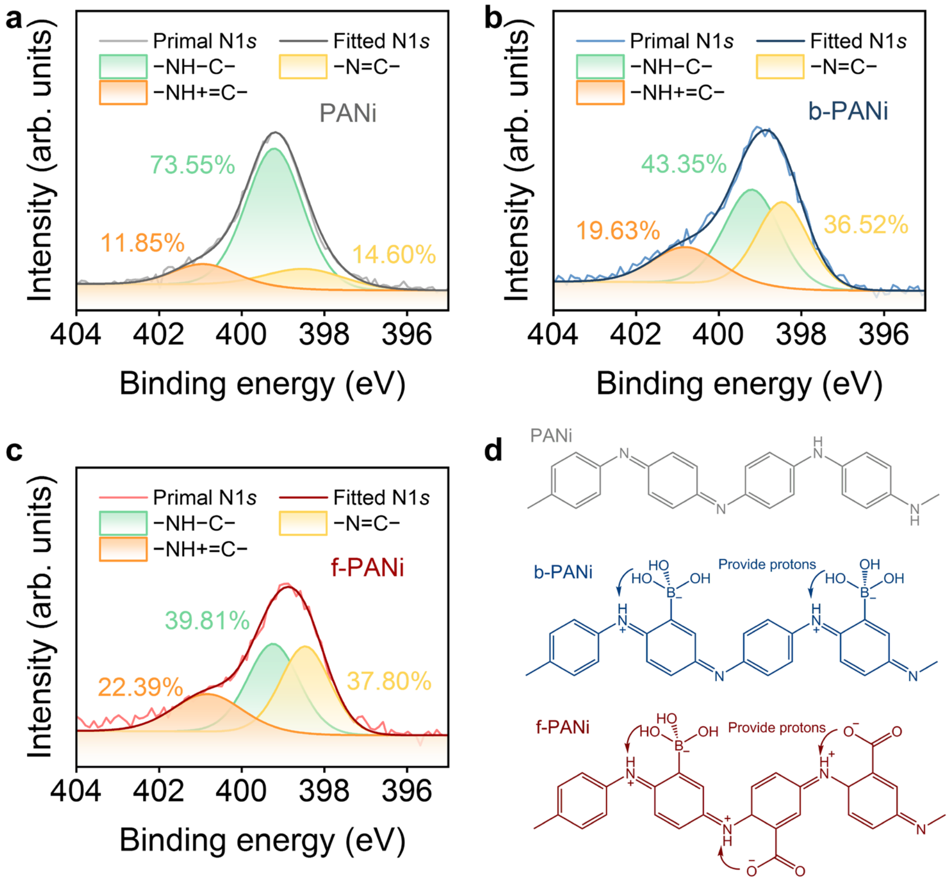

Supplementary Fig. 2 | XPS of N1*s* for PANi (**a**), b-PANi (**b**), and f-PANi (**c**). (**d**) The doping structures of conducting polymers.

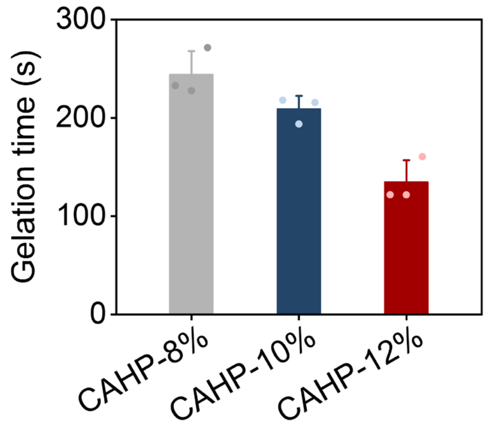

Supplementary Fig. 3 | The gelation time of the CAHP is required for the precursor solution of CAHP to undergo the sol-gel transition (n = 3 independent experiments). Data are presented as the mean ± standard deviation.

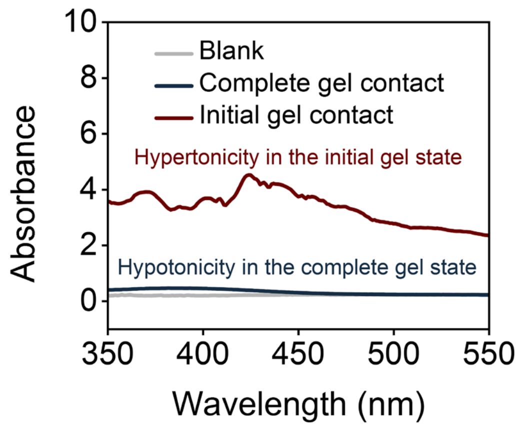

Supplementary Fig. 4 | The absorbance of f-PANi infiltrated the gelatin substrate in the range of 350 to 550 nm.

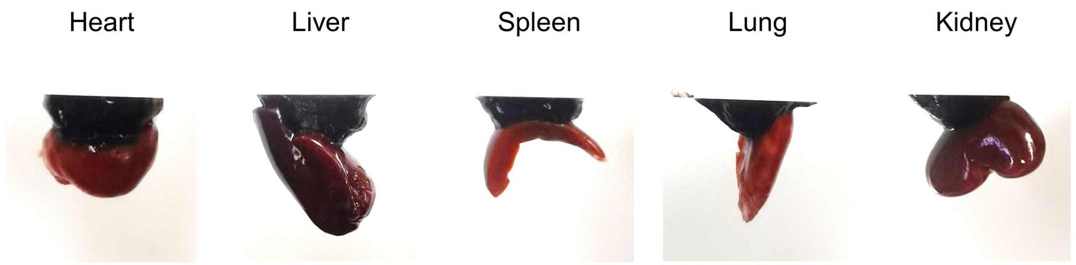

Supplementary Fig. 5 | Photographs of CAHP adhesion to various tissues.

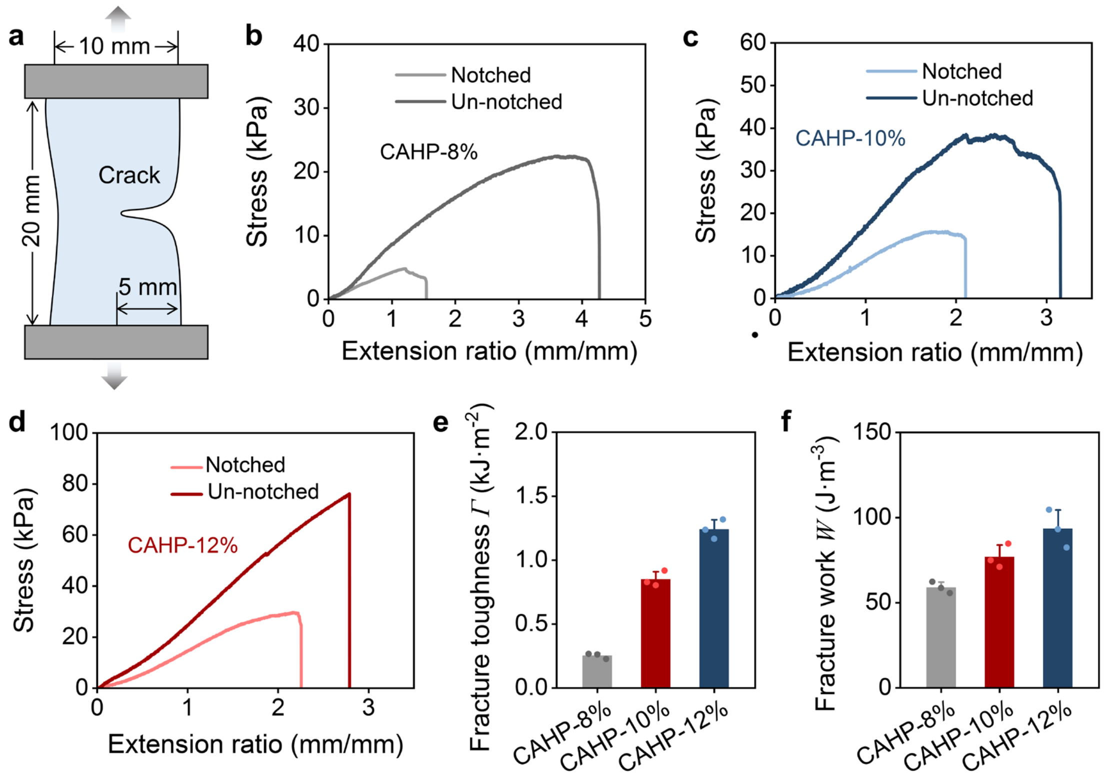

Supplementary Fig. 6 | The fracture energy of the CAHPs. (**a**) Measurement of the fracture energy by the single-edge notched tensile method. Tensile curves of CAHP-8% (**b**), CAHP-10% (**c**), and CAHP-12% (**d**) with and without a notch. The fracture toughness Γ (**e**) and fracture work W (**f**) of CAHPs (n = 3 independent experiments). Data are presented as the mean ± standard deviation in (**e**, **f**).

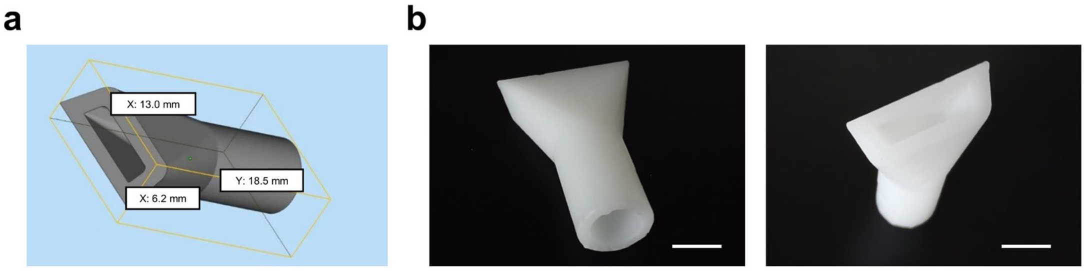

Supplementary Fig. 7 | Design diagram and photos of a custom applicator. (**a**) Three-dimensional design drawing of an applicator with a length of 18 mm, a width of 13 mm and a height of 6.2 mm. The applicator can be connected to a syringe, allowing the hydrogel precursor to be shaped into sheets of 1 mm thickness and 10 mm width. (**b**) Photographs showing the front (left) and back (right) ends of the applicator. Scale bars, 5 mm.

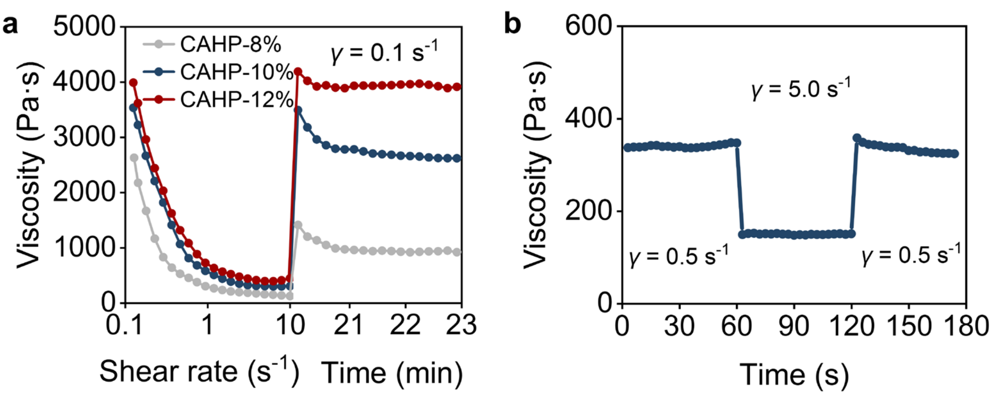

Supplementary Fig. 8 | The shear thinning and self-healing properties of the CAHP. (**a**) Viscosity change of the CAHP during continuous loading (0.1 s^{-1}–10 s^{-1}) and sudden unloading (0.1 s^{-1}). (**b**) Viscosity change of CAHP-12% subjected to step shear between 0.5 s^{-1} and 5 s^{-1} shear rates (γ).

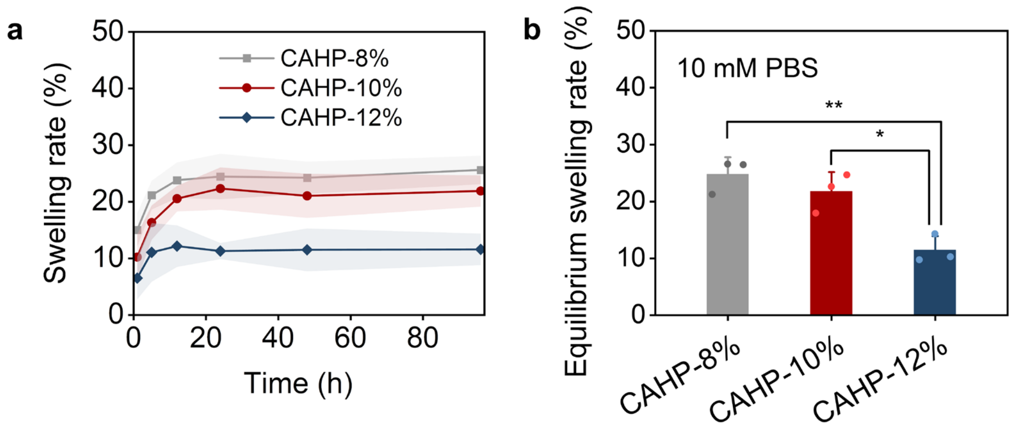

Supplementary Fig. 9 | (**a**) The swelling performance of CAHP in PBS solution for 96 h (*n* = 3 independent experiments). (**b**) Equilibrium swelling rate of CAHPs (*n* = 3 independent experiments). Data are presented as the mean ± standard deviation in (**a**, **b**) and were analyzed using one-way ANOVA with Tukey's post hoc test in (**b**), *$p < 0.05$, ** $p < 0.01$. **b** $p = 4.26 \times 10^{-3}$ (CAHP-8% vs CAHP-12%); $p = 0.0138$ (CAHP-10% vs CAHP-12%).

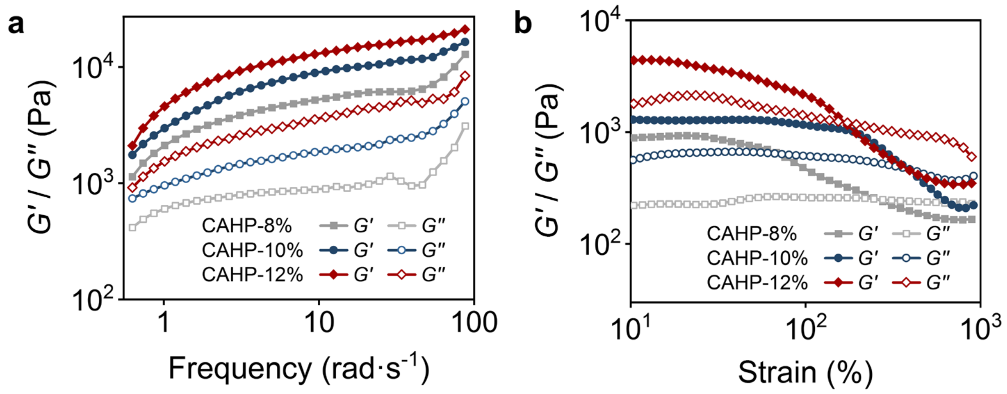

Supplementary Fig. 10 | Rheological properties of the CAHP. (**a**) Rheological frequency sweep tests over a frequency range from 0.5 to 100 rad·s^{-1}. (**b**) Rheological strain sweep tests over a strain range from 10% to 1000%. *G′*: storage modulus; *G″*: loss modulus.

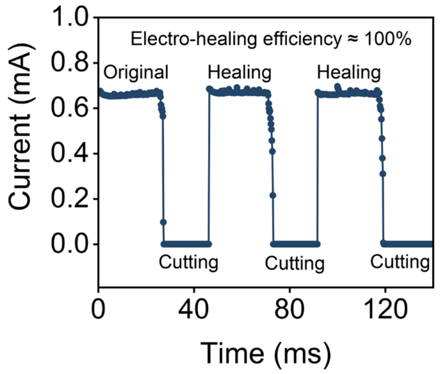

Supplementary Fig. 11 | The current changes of the electrical self-healing process in the disconnect-reconnect cycle of CAHP.

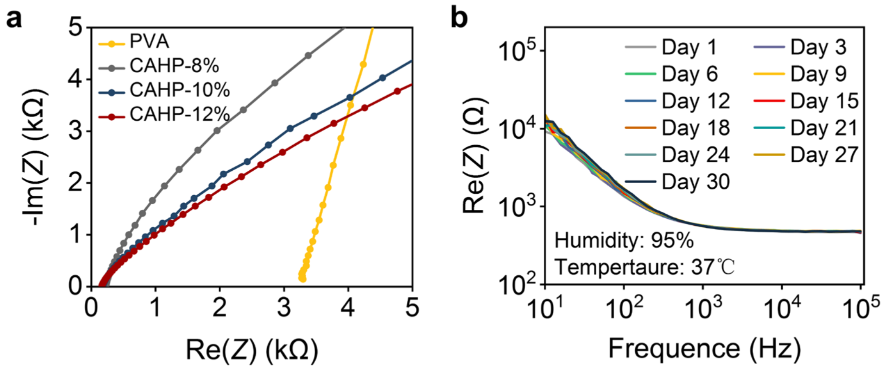

Supplementary Fig. 12 | Electrochemical impedance (*Z*) of the CAHP. (**a**) Nyquist plot of the PVA hydrogel and CAHPs. (**b**) The electrochemical impedance of CAHP-12% immersed in PBS solution at 37 °C and 95% humidity for 30 days.

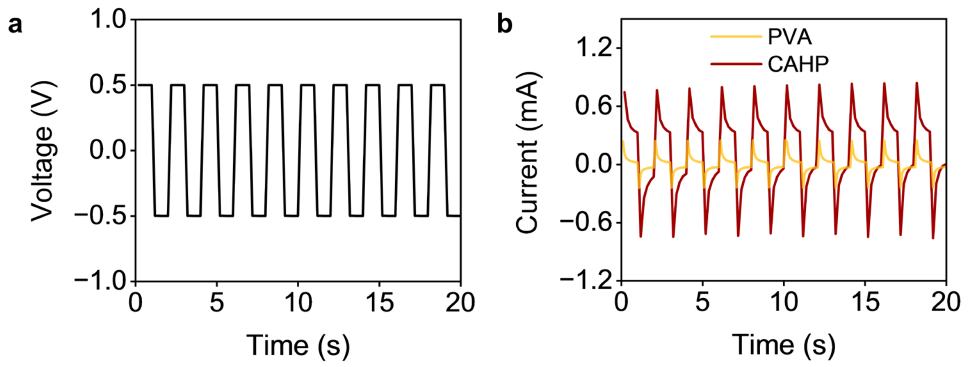

Supplementary Fig. 13 | The charge injection capacity of the PVA hydrogel and CAHP. (**a**) Cyclic bidirectional pulses of ±0.5 V. (**b**) Charge injection curves of the PVA hydrogel and CAHP.

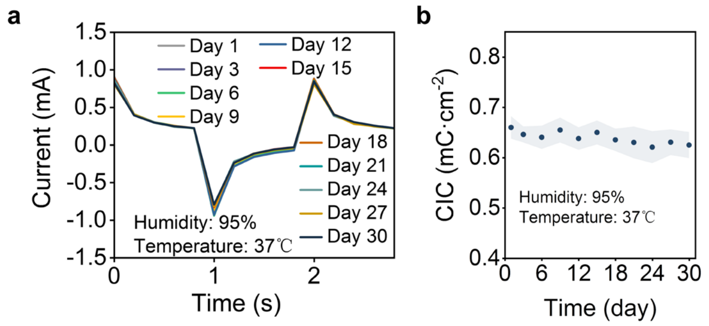

Supplementary Fig. 14 | The electrochemical stability of CAHP. (**a**) Charge injection curves of CAHP-12% immersed in PBS solution at 37 °C and 95% humidity for 30 days. (**b**) Charge injection capability (CIC) of CAHP-12% within 30 days (n = 3 independent experiments). Data are presented as the mean ± standard deviation in (**b**).

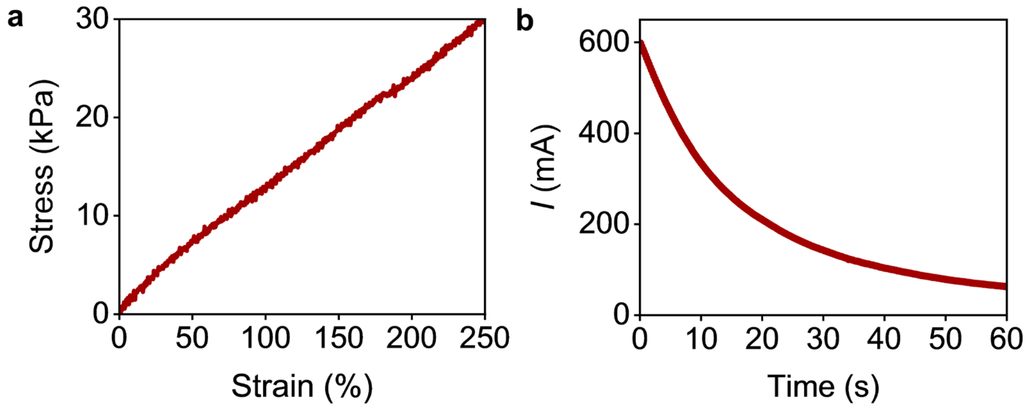

Supplementary Fig. 15 | Stress−strain curves (**a**) and deformation-induced current change curves (**b**) of CAHP-12% in uniaxial tension.

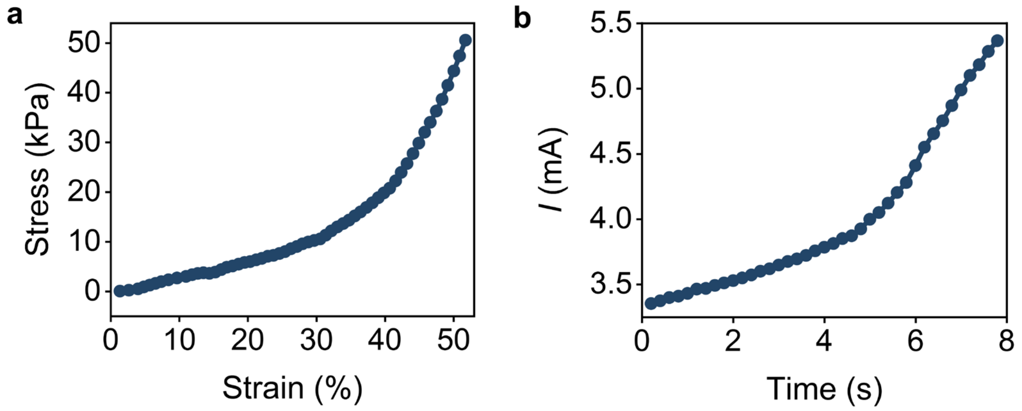

Supplementary Fig. 16 | Stress−strain curves (**a**) and deformation-induced current change curves (**b**) of CAHP-12% under uniaxial compression.

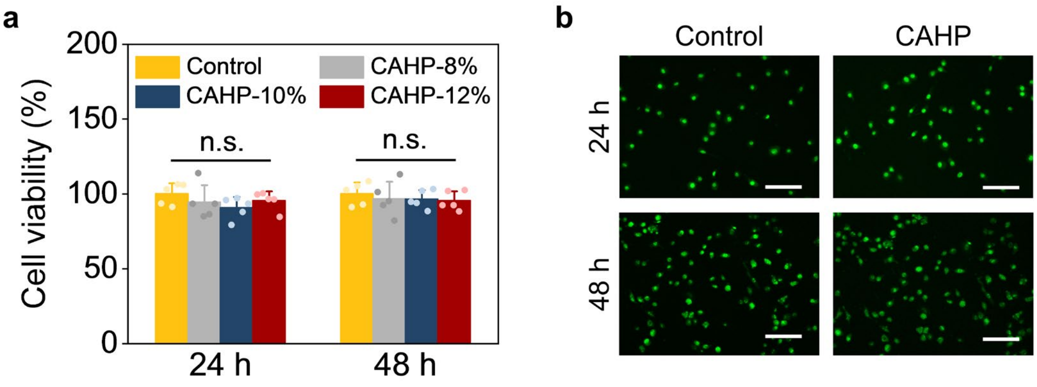

Supplementary Fig. 17 | The cytocompatibility of the CAHP. MTT-detected cell viability (**a**) and acridine orange staining (**b**) of L929 cells with the CAHP extracts (n = 5 independent experiments). The measurements in (**b**) were repeated five times independently with similar results. Scale bars, 100 μm. Data are presented as the mean ± standard deviation and were analyzed using one-way ANOVA with Tukey's post hoc test in (**a**), n.s.: no significant difference at $p > 0.05$. **a** 24 h: $p = 0.379$ (Control vs CAHP-8%); $p = 0.075$ (Control vs CAHP-10%); $p = 0.315$ (Control vs CAHP-12%). 48 h: $p = 0.610$ (Control vs CAHP-8%); $p = 0.435$ (Control vs CAHP-10%); $p = 0.337$ (Control vs CAHP-12%).

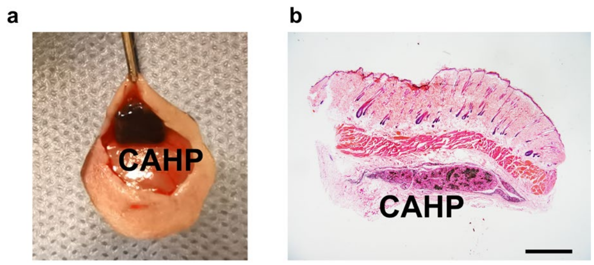

Supplementary Fig. 18 | Photographs (**a**) and hematoxylin-eosin staining images (**b**) of CAHP subcutaneously implanted in mice for 7 days. The measurements in (**b**) were repeated three times independently with similar results. Scale bars, 1 mm.

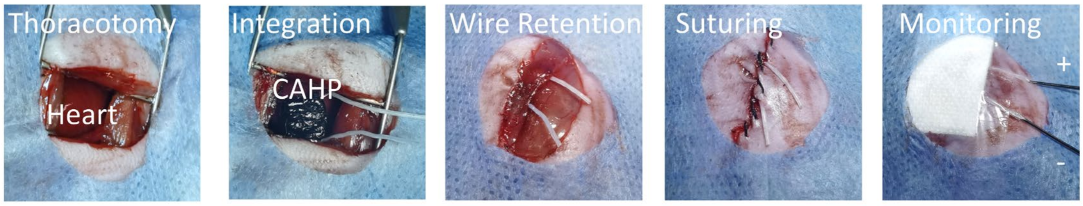

Supplementary Fig. 19 | The CAHP installation procedure included thoracotomy, CAHP fixation, wire retention, suturing, and attachment of monitoring electrodes.

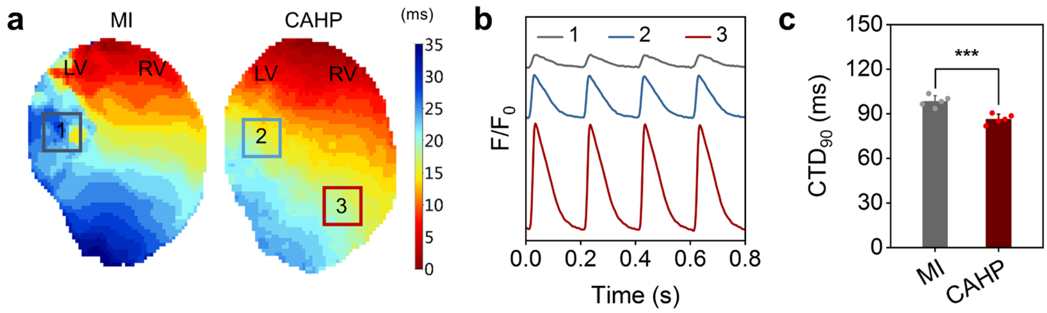

Supplementary Fig. 20 | Isolated hearts in the MI and CAHP groups were perfused with fluorescent dyes of calcium transients and electrically stimulated at the apex. (**a**) A high-speed camera captured the fluorescence signal to plot the activation time heatmap of the electrical signal propagation throughout the infarcted and healthy regions. LV: left ventricle; RV: right ventricle. (**b**) Relative fluorescence intensity (F/F_0) of calcium transients in the corresponding positions (1: Infarct region in the MI group; 2: Infarct region in the CAHP group; 3: Healthy region.) in the heatmap. (**c**) Time taken to restore 90% of calcium flow (CTD_{90}) in the MI and CAHP groups (n = 5 animals). Data are presented as the mean ± standard deviation and were analyzed using one-way ANOVA with Tukey's post hoc test in (**c**), ***$p < 0.001$. **c** $p = 7.44 \times 10^{-4}$ (MI vs CAHP).

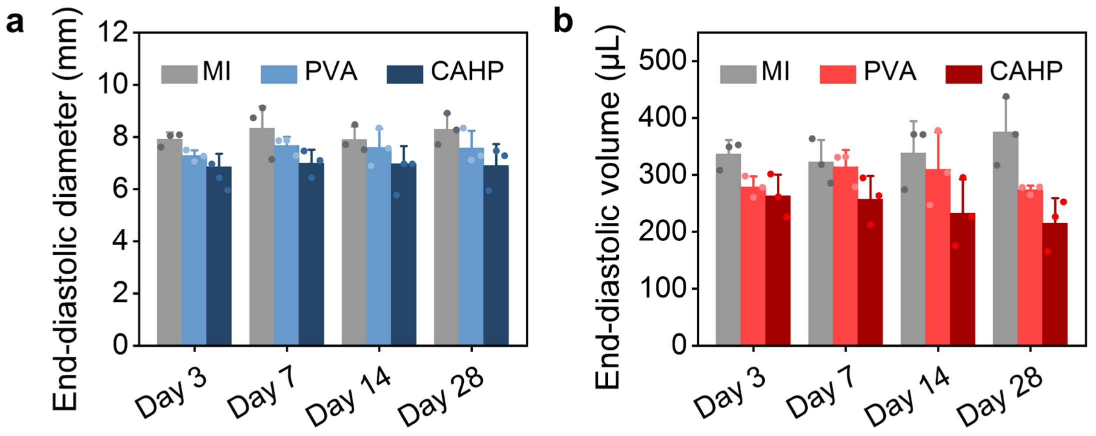

Supplementary Fig. 21 | End-diastolic diameters (**a**) and volumes (**b**) of hearts in the MI, PVA, and CAHP groups within 28 days (*n* = 3 animals). Data are presented as the mean ± standard deviation.

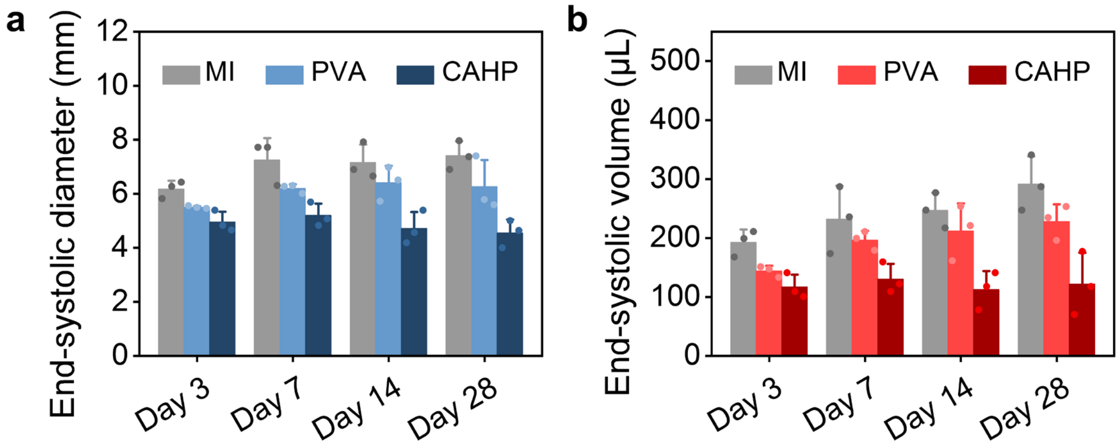

Supplementary Fig. 22 | End-systolic diameters (**a**) and volumes (**b**) of hearts in the MI, PVA, and CAHP groups within 28 days (*n* = 3 animals). Data are presented as the mean ± standard deviation.

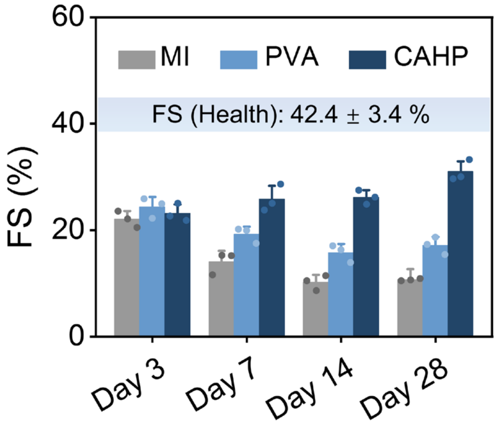

Supplementary Fig. 23 | Fractional shortening (FS) of hearts in the MI, PVA, and CAHP groups within 28 days ($n = 3$ animals). Data are presented as the mean ± standard deviation.

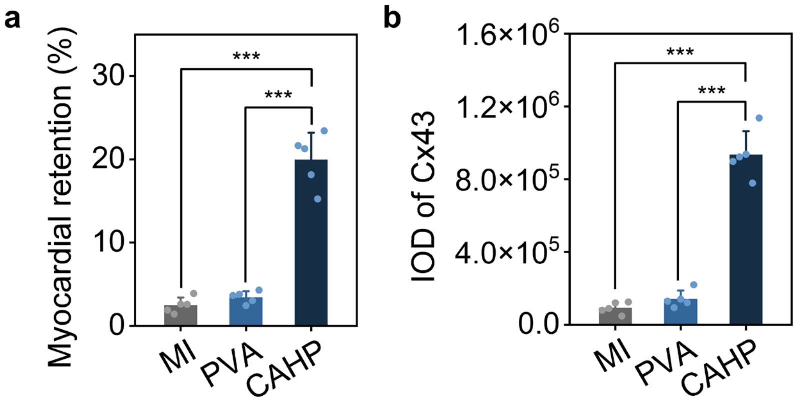

Supplementary Fig. 24 | Statistical analysis of myocardial retention (**a**) and integrated optical density (IOD) of Cx43 (**b**) in cardiac infarcted regions in the MI, PVA, and CAHP groups ($n = 5$ animals). Data are presented as the mean ± standard deviation and were analyzed using one-way ANOVA with Tukey's post hoc test, ***$p < 0.001$. **a** $p = 2.83 \times 10^{-6}$ (MI vs CAHP); $p = 3.85 \times 10^{-6}$ (PVA vs CAHP). **b** $p = 6.00 \times 10^{-7}$ (MI vs CAHP); $p = 1.22 \times 10^{-6}$ (PVA vs CAHP).

Supplementary Table 1 | Semiquantitative elemental percent in f-PANi

Name	Peak (eV)	Percent %
O1*s*	531.8	26.89
N1*s*	398.4	9.75
C1*s*	284.8	60.51
B1*s*	188.0	2.85

Supplementary Table 2 | Component content of the CAHPs

Hydrogel	Solid content (wt%)	f-PANi (wt%)	PVA (wt%)
CAHP-8%	8	4	4
CAHP-10%	10	5	5
CAHP-12%	12	6	6
