# Supplementary material for: Chronological adhesive cardiac patch for synchronous mechanophysiological monitoring and electrocoupling therapy
Source: Nat Commun. 2023 Oct 6;14:6226. doi: 10.1038/s41467-023-42008-9 (PMC10558550; doi:10.1038/s41467-023-42008-9)
Supplement: Supplementary file 2 — Reporting Summary [file 41467_2023_42008_MOESM2_ESM.pdf]

nature portfolio

Corresponding author(s): Junjie Li

Last updated by author(s): Aug 28, 2023

Reporting Summary

Nature Portfolio wishes to improve the reproducibility of the work that we publish. This form provides structure for consistency and transparency in reporting. For further information on Nature Portfolio policies, see our Editorial Policies and the Editorial Policy Checklist.

Statistics

For all statistical analyses, confirm that the following items are present in the figure legend, table legend, main text, or Methods section.

n/a	Confirmed	
☐	☒	The exact sample size (n) for each experimental group/condition, given as a discrete number and unit of measurement
☐	☒	A statement on whether measurements were taken from distinct samples or whether the same sample was measured repeatedly
☐	☒	The statistical test(s) used AND whether they are one- or two-sided<br>*Only common tests should be described solely by name; describe more complex techniques in the Methods section.*
☒	☐	A description of all covariates tested
☒	☐	A description of any assumptions or corrections, such as tests of normality and adjustment for multiple comparisons
☐	☒	A full description of the statistical parameters including central tendency (e.g. means) or other basic estimates (e.g. regression coefficient) AND variation (e.g. standard deviation) or associated estimates of uncertainty (e.g. confidence intervals)
☐	☒	For null hypothesis testing, the test statistic (e.g. F, t, r) with confidence intervals, effect sizes, degrees of freedom and P value noted<br>*Give P values as exact values whenever suitable.*
☒	☐	For Bayesian analysis, information on the choice of priors and Markov chain Monte Carlo settings
☒	☐	For hierarchical and complex designs, identification of the appropriate level for tests and full reporting of outcomes
☒	☐	Estimates of effect sizes (e.g. Cohen's d, Pearson's r), indicating how they were calculated

Our web collection on statistics for biologists contains articles on many of the points above.

Software and code

Policy information about availability of computer code

Data collection	Fourier transform infrared spectroscopy (Nicolet 6700, Thermo Fisher), X-ray photoelectron spectroscopy (K-Alpha+, Thermo Fisher), Ultraviolet–visible spectroscopy (Evolution201, Thermo Fisher), Zetasizer (Nano ZS, Malvern), Contact angle instrument (JC200FM, Zhongchen Digital), Scanning electronic microscopy (S-4800, Hitachi), Enzyme immunoassay (Infinite M Nano+, Tecan), Fluorescence microscope (Eclipse Ts2, Nikon), Rheometer (Viscotester iQ, Thermo Fisher), Universal machine (WDW-05, Si Pai), Electrochemical station (Vertex C, Ivium), Biosignal processing system (PowerLab, AD Instruments), Echocardiography (Vevo 2100, VisualSonics), Fluorescent mapping system (OMSC801, MappingLab), Confocal platform (Andor Dragonfly, Oxford Instruments).
Data analysis	Origin 2023 software and Microsoft Excel 2016 were used for data plotting and statistical analysis. Image J software was used for quantitative imaging analyses. Avantage software was used for XPS analysis. LabChart 7 software was used for ECG analysis. OMapScope 5 software was used to analyze electrophysiology mapping.

For manuscripts utilizing custom algorithms or software that are central to the research but not yet described in published literature, software must be made available to editors and reviewers. We strongly encourage code deposition in a community repository (e.g. GitHub). See the Nature Portfolio guidelines for submitting code & software for further information.

Data

Policy information about availability of data

All manuscripts must include a data availability statement. This statement should provide the following information, where applicable:

- Accession codes, unique identifiers, or web links for publicly available datasets
- A description of any restrictions on data availability
- For clinical datasets or third party data, please ensure that the statement adheres to our policy

All data supporting the findings of this study are available within the paper and its supplementary information or from the corresponding authors on request. Source data are provided with this paper.

Research involving human participants, their data, or biological material

Policy information about studies with human participants or human data. See also policy information about sex, gender (identity/presentation), and sexual orientation and race, ethnicity and racism.

Reporting on sex and gender	This work did not involve human research.
Reporting on race, ethnicity, or other socially relevant groupings	This work did not involve human research.
Population characteristics	This work did not involve human research.
Recruitment	This work did not involve human research.
Ethics oversight	This work did not involve human research.

Note that full information on the approval of the study protocol must also be provided in the manuscript.

Field-specific reporting

Please select the one below that is the best fit for your research. If you are not sure, read the appropriate sections before making your selection.

☒ Life sciences ☐ Behavioural & social sciences ☐ Ecological, evolutionary & environmental sciences

For a reference copy of the document with all sections, see nature.com/documents/nr-reporting-summary-flat.pdf

Life sciences study design

All studies must disclose on these points even when the disclosure is negative.

Sample size	Details regarding the sample size of all experiments are provided in the Methods section and figure legends. For material characterization, we determined the sample size based on previous experience and other publications (Nature Communications 2022, 13, 7666; Nature Communications 2019, 10, 2060). Each sample was tested at least three times to ensure that they were sufficient for statistical comparison between different groups. The sample was randomly chosen to be determined.<br>Power analysis was used to evaluate the sample size in the animal experiments. According to the mechanical compensation and electrical coupling studies of myocardial infarction by polyaniline-based hydrogel patches in our previous study (ACS Nano 2022, 16, 16234), there was a significant restoration of myocardial pulsatile and electrophysiological functions. Therefore, the significance level was set as 0.05, the power was set as 0.8, the difference was set as 0.99, and the sample size was calculated as 3 rats per group. Animal experiments were performed on 5 rats per group, and mechanophysiological monitoring, echocardiography, and electrocardiography were performed on three randomly selected rats in each group at different times.
Data exclusions	No data were excluded from this study.
Replication	All experiments were performed with independent replicates as described in the figure legends.
Randomization	All samples were randomly allocated into experimental groups.
Blinding	Investigators were blinded to group allocation during data collection and analysis.

Reporting for specific materials, systems and methods

We require information from authors about some types of materials, experimental systems and methods used in many studies. Here, indicate whether each material, system or method listed is relevant to your study. If you are not sure if a list item applies to your research, read the appropriate section before selecting a response.

Materials & experimental systems

n/a	Involved in the study
	☒ Antibodies
	☒ Eukaryotic cell lines
☒	Palaeontology and archaeology
	☒ Animals and other organisms
☒	Clinical data
☒	Dual use research of concern
☒	Plants

Methods

n/a	Involved in the study
☒	ChIP-seq
☒	Flow cytometry
☒	MRI-based neuroimaging

Antibodies

Antibodies used

Primary antibodies: Anti-Alpha-cardiac Actin (α-Actin; Mouse Monoclonal; Huabio; Catalog No. M1206-1); Anti-Cardiac Troponin T (cTnT; Rabbit Monoclonal; Huabio; Catalog No. ET1610-51); Anti-Connexin 43 (Cx43; Rabbit Polyclonal; ABclonal; Catalog No. A11752); Anti-Alpha Smooth Muscle Actin Antibody (α-SMA; Mouse Monoclonal; BioLegend; Catalog No. 904601); Wheat Germ Agglutinin (WGA; Alexa Fluor™ 594; Catalog No. W11262). Secondary antibodies:Donkey anti-Rabbit IgG (H+L) Highly Cross-Adsorbed Secondary Antibody (Alexa Fluor™ 594; Thermo Fisher; Catalog No. A21207); Donkey anti-Mouse IgG (H+L) Highly Cross-Adsorbed Secondary Antibody (Alexa Fluor™ 488; Thermo Fisher; Catalog No. A21202).

Validation

Anti-Alpha-cardiac Actin Mouse Monoclonal Antibody [33-32] (M1206-1; Huabio; 1:200): Species Reactivity: Human, Mouse, Rat, Zebrafish; Application: WB, IF, IHC-P.
Anti-Cardiac Troponin T Recombinant Rabbit Monoclonal Antibody [SC64-03] (ET1610-51; Huabio; 1:200): Species Reactivity: Human, Mouse, Rat; Application: WB, IHC-P, IF, FC.
Anti-Connexin 43 Rabbit Polyclonal Antibody (A11752; ABclonal; 1:100): Species Reactivity: Human, Mouse, Rat; Application: WB, IHC-P, IF, ICC.
Anti-Alpha Smooth Muscle Actin Mouse Monoclonal Antibody (904601; BioLegend; 1:200); Species Reactivity: Human, Mouse, Rat, Cow, Chicken; Application: WB, IHC-P, IF/ICC.
Wheat Germ Agglutinin (WGA; Alexa Fluor™ 594; Catalog No. W11262; 1:400); Species: mammalian cells; Application: IF, IHC, FC
Donkey anti-Rabbit IgG (H+L) Highly Cross-Adsorbed Secondary Antibody (Alexa Fluor™ 594; A21207; Thermo Fisher; 1:500); Species Reactivity: Rabbit; Application: IF/ICC.
Donkey anti-Mouse IgG (H+L) Highly Cross-Adsorbed Secondary Antibody (Alexa Fluor™ 488; A21202; Thermo Fisher; 1:500); Species Reactivity: Mouse; Application: IF/ICC.

Eukaryotic cell lines

Policy information about cell lines and Sex and Gender in Research

Cell line source(s)

L929 cell lines were purchased from iCell Bioscience (Shanghai, China) and derived from mice. The cell line source did not take gender into account.

Authentication

L929 cell lines were not authenticated by the investigators in this paper. They were purchased from the company.

Mycoplasma contamination

L929 cell lines were not tested for mycoplasma contamination.

Commonly misidentified lines
(See ICLAC register)

No commonly misidentified cell lines were used in the study.

Animals and other research organisms

Policy information about studies involving animals; ARRIVE guidelines recommended for reporting animal research, and Sex and Gender in Research

Laboratory animals

Male Sprague-Dawley rats (SD rats, 8 weeks old) and male Kunming mice (8 weeks old) were used for all our experiments.

Wild animals

This study did not involve wild animals.

Reporting on sex

This study did not apply to only one sex. The study design and methods did not take sex into consideration. All male animals were used in this study, and sex differences were not considered. Animal experiments were not considered for sex analysis because myocardial structure and functions in the MI model and inflammatory responses in subcutaneous encapsulation were not related to sex.

Field-collected samples

This study did not involve samples collected from the field.

Ethics oversight

The animal experiments were approved by and performed according to the guidelines of the Institutional Animal Care and Use Committee of the North China University of Science and Technology.

Note that full information on the approval of the study protocol must also be provided in the manuscript.
