# Supplementary material for: Chronological adhesive cardiac patch for synchronous mechanophysiological monitoring and electrocoupling therapy
Source: Nat Commun. 2023 Oct 6;14:6226. doi: 10.1038/s41467-023-42008-9 (PMC10558550; doi:10.1038/s41467-023-42008-9)
Supplement: Supplementary file 3 — Description of Additional Supplementary Files [file 41467_2023_42008_MOESM3_ESM.pdf]

Title: Supplementary Movie 1
Description: Conducting polymer dispersion permeation through the filter membrane.

Title: Supplementary Movie 2
Description: Rapid selfhealing of the CAHP.
